# Updates on preoperative planning, limb deformity analysis and surgical correction for the growing children: EPOS Meeting Marseille 2015 proceedings

**DOI:** 10.1007/s11832-016-0795-8

**Published:** 2016-11-08

**Authors:** Eitan Segev

**Affiliations:** 1 Department of Pediatric Orthopaedics, Dana Children’s Hospital, Tel Aviv Sourasky Medical Center, Tel Aviv, Israel; 2 Sackler Faculty of Medicine, Tel Aviv University, Tel Aviv, Israel

**Keywords:** Limb deformity analysis, Length difference, Prediction, Surgical correction, Follow-up

## Abstract

Successful deformity correction depends on establishing the aetiology of the deformity. Clinical examination, additional laboratory tests and consultation with other experts may be needed to complete the workup. Imaging studies should include full-length standing X-rays in all relevant planes, and additional imaging modalities like computed tomography (CT) and magnetic resonance imaging (MRI) may add information on bone morphology and growth plates’ anatomy. Based on the data, analysis of the deformity and length differences is performed, followed by prediction of deformities at skeletal maturity. The patients need to be followed up on a regular basis and repeat analysis should be done to improve the accuracy of prediction for final limb length difference. Limb deformity and lengthening correction plans are drawn and updated during follow-up, to achieve straight and equal lower limbs at maturity. Timely surgical procedures are performed using appropriate techniques and the most modern technologies available. These principles are discussed and demonstrated with case examples.

## Introduction

Requirements for successful limb length difference and deformity correction can be described in a stepwise procedure:

1. Identify aetiology of the deformity, obtain medical and family history.
2. Perform clinical examination with general and specific findings.
3. Obtain metabolic, endocrine profile when relevant.
4. Using properly calibrated, full-length standing X-rays, analyse deformities in all relevant planes using the healthy side for reference or established anatomical and mechanical standard measurements.
5. Predict final length difference and deformity, calculate adult height of the patient using an Anderson’s data-based application.
6. Plan for straight equal lower limbs at maturity—stage the treatment.
7. Follow up and properly time the surgery.
8. Use the simplest possible and appropriate surgical techniques.
9. Deal promptly with complications.
10. Follow up to skeletal maturity.

## Preoperative planning

**Fig. 1:**
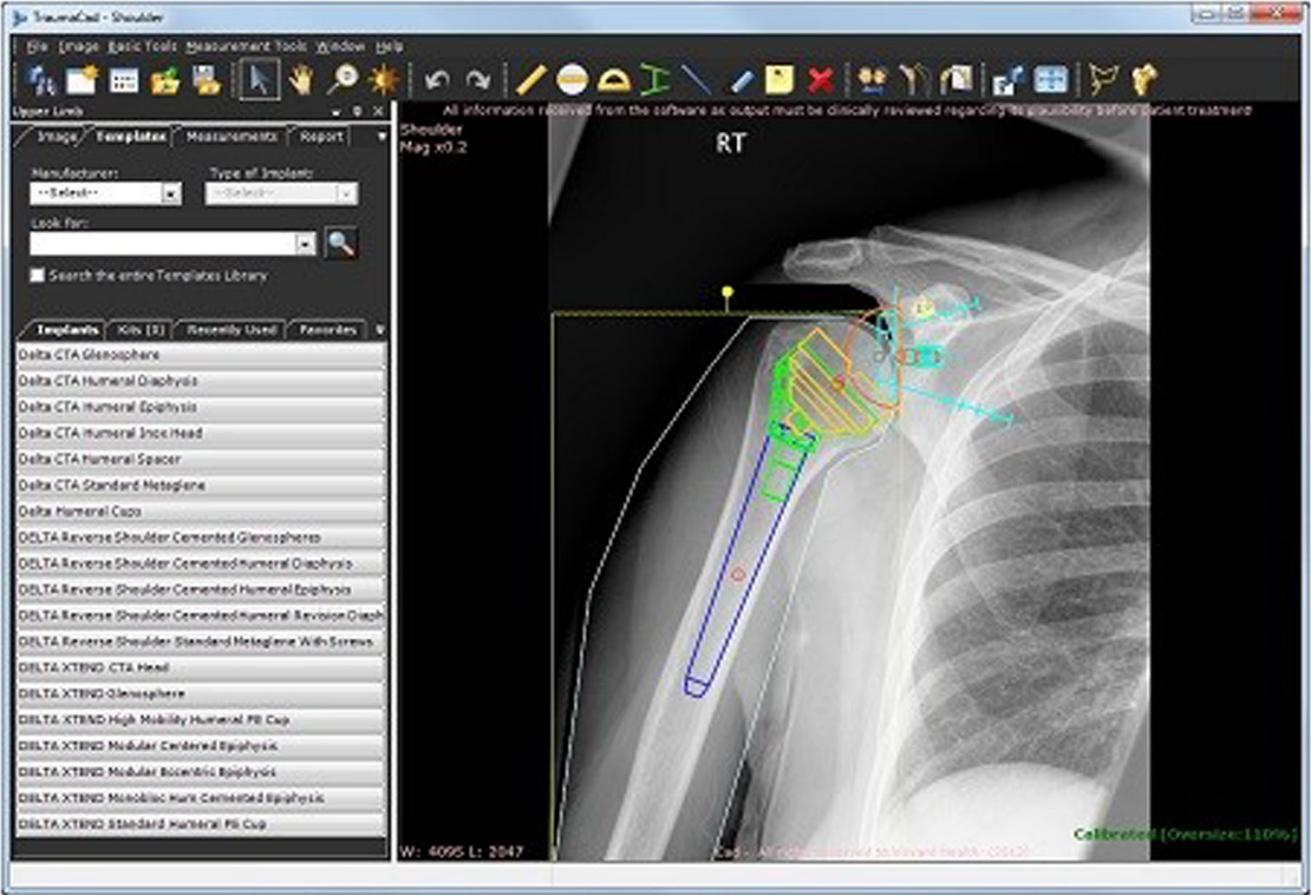
The TraumaCad software for preoperative planning, limb length difference and deformity analysis is a comprehensive tool for planning

**Fig. 2:**
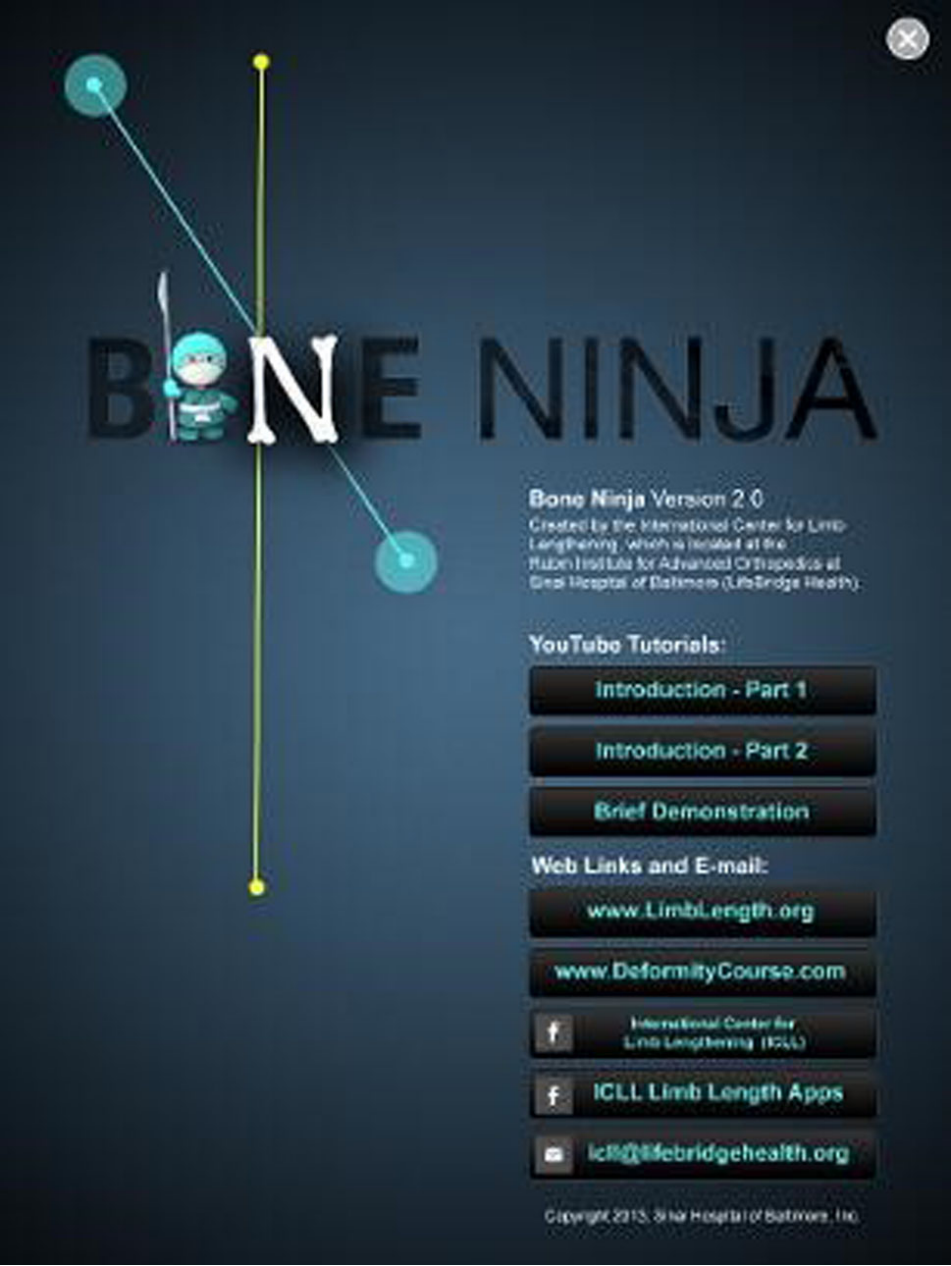
The Baltimore Center Bone Ninja is an iPad or iPhone application

**Fig. 3:**
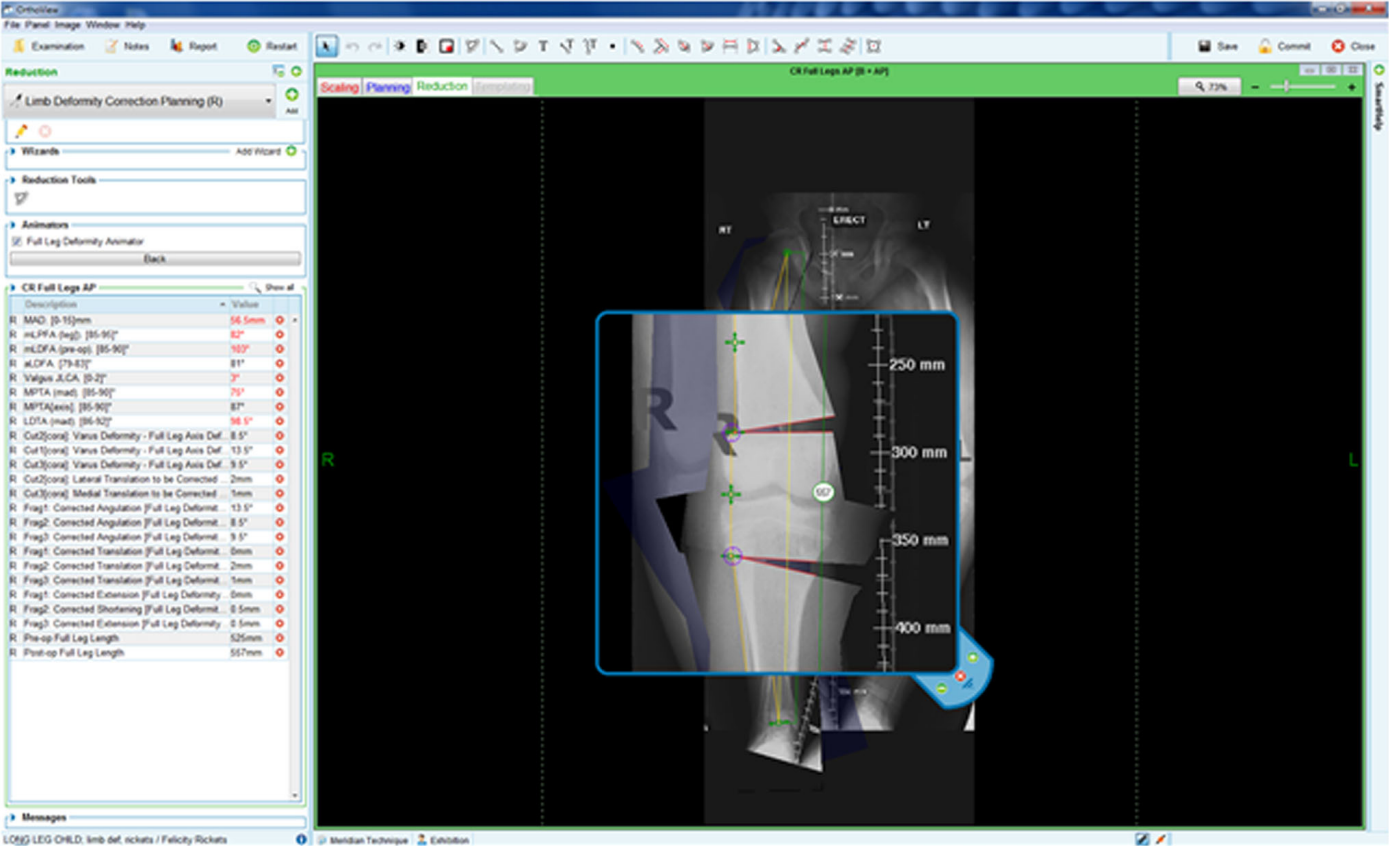
Digital preoperative planning for limb deformity assessment and correction: OrthoView software

Having completed the history recording and performed relevant clinical examination, a deformity analysis digital software tool like TraumaCad [1], Baltimore Bone Ninja or OrthoView is used to establish the current pathology (Figs. 1, 2 and 3).

**Fig. 4:**
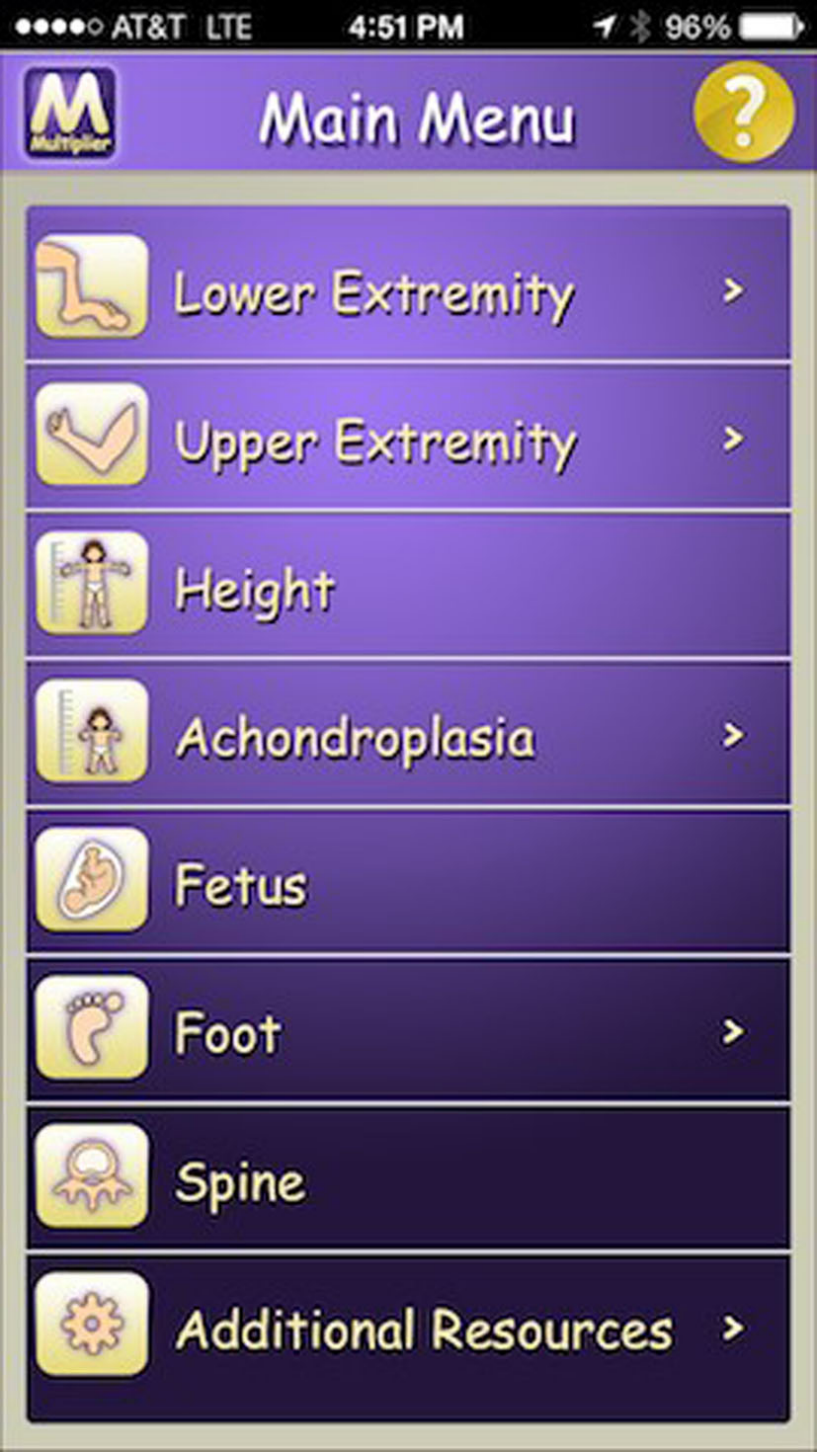
Baltimore application

**Fig. 5:**
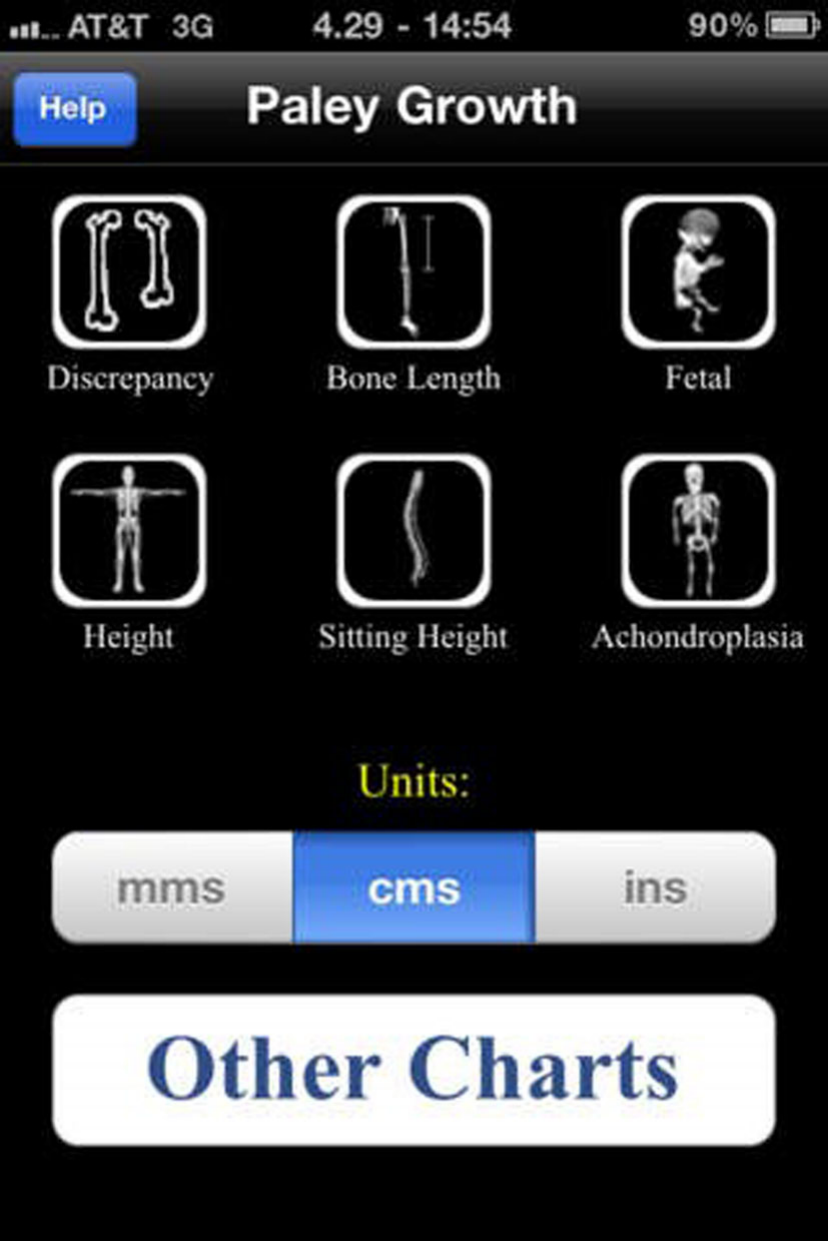
Paley Growth application

The prediction of the final height, final limb’s length and difference can be calculated using data published in the literature or other digital applications, like the Baltimore or the Paley Growth applications, based on the Anderson tables and the Baltimore multiplier (Figs. 4 and 5). Most of these applications are available on the Apple store.

Based on the data accumulated, a limb deformity correction and length equalisation plan is presented to the family. The surgical and follow-up plan is carried out with the aim of achieving these goals by skeletal maturity.

We will present case examples to demonstrate these recommendations.

## Case example 1

**Fig. 6:**
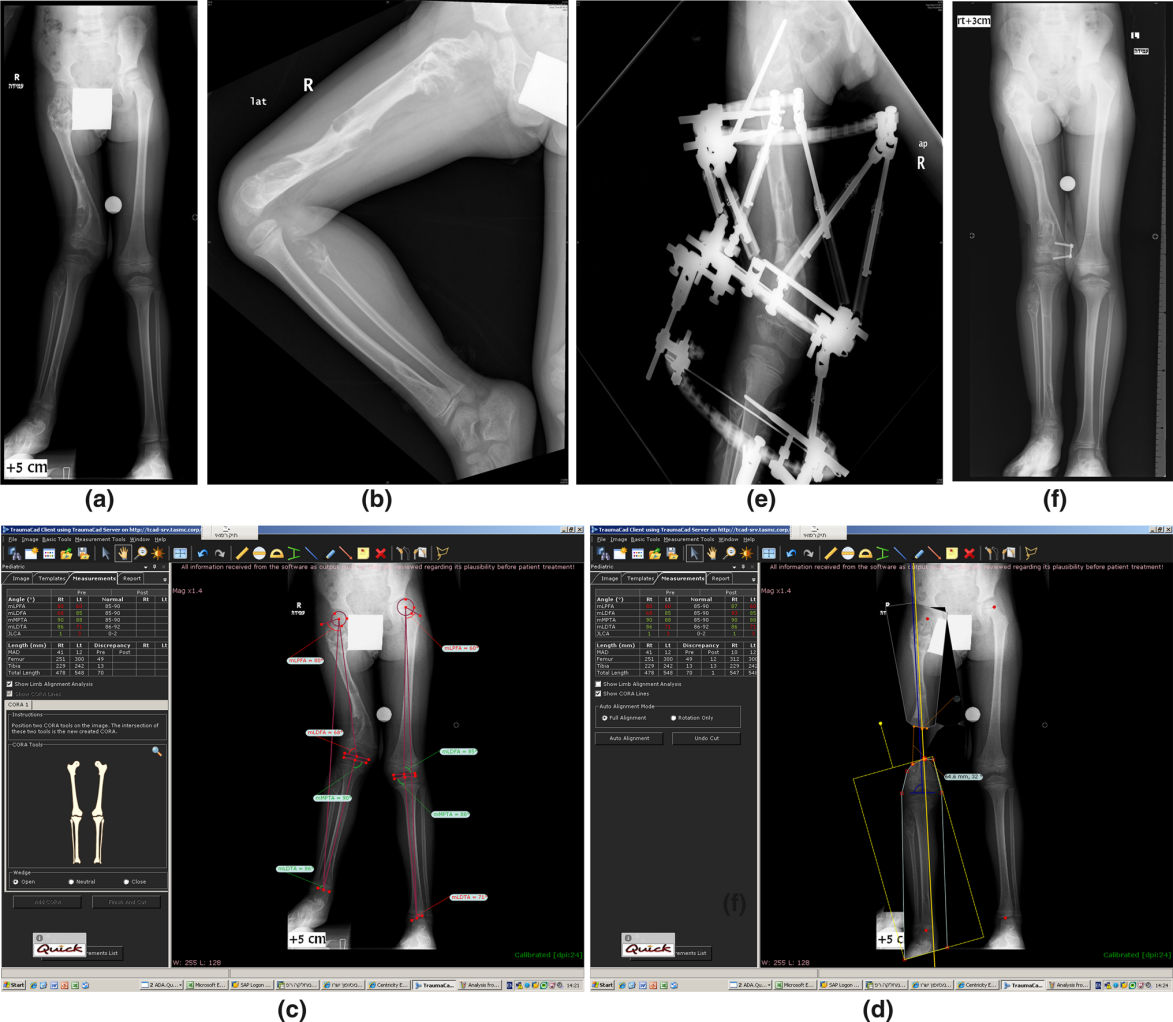
Case example 1

Male, age 5.6 years, diagnosis of Ollier disease—right lower limb more affected. Current clinical shortening is 70 mm (Fig. 6a, b).

Deformity and length are analysed in a frontal plane using the TraumaCad software. The shortening of 70 mm is mainly femoral, associated to a distal femoral valgus (Fig. 6c).

The predictions of the final length difference and the age for epiphysiodesis, using the Excel spreadsheet software package, are based on the Anderson tables and Baltimore multiplier [2].

### Considerations

In bone disease (Ollier disease), the cysts are producing bone. The bone can hold external fixation pins. The deformity of the distal femur is in valgus. The predicted shortening is 11.8 cm.

We plan to lengthen 60 mm due to increased complications with bigger lengthening. We will use an external fixator and lengthen via the femoral CORA to correct the valgus deformity in the same procedure. In the future, the patient will need another lengthening procedure or a contralateral epiphysiodesis.

The simulation of the deformity correction with 60 mm lengthening via the CORA is obtained using the TraumaCad software (Fig. 6d). The lengthening with Taylor Spatial Frame is performed with a protection of the proximal femoral neck (Fig. 6e).

One year post frame removal, the recurrent valgus is managed by an eight plate (Fig. 6f).

## Case example 2

**Fig. 7:**
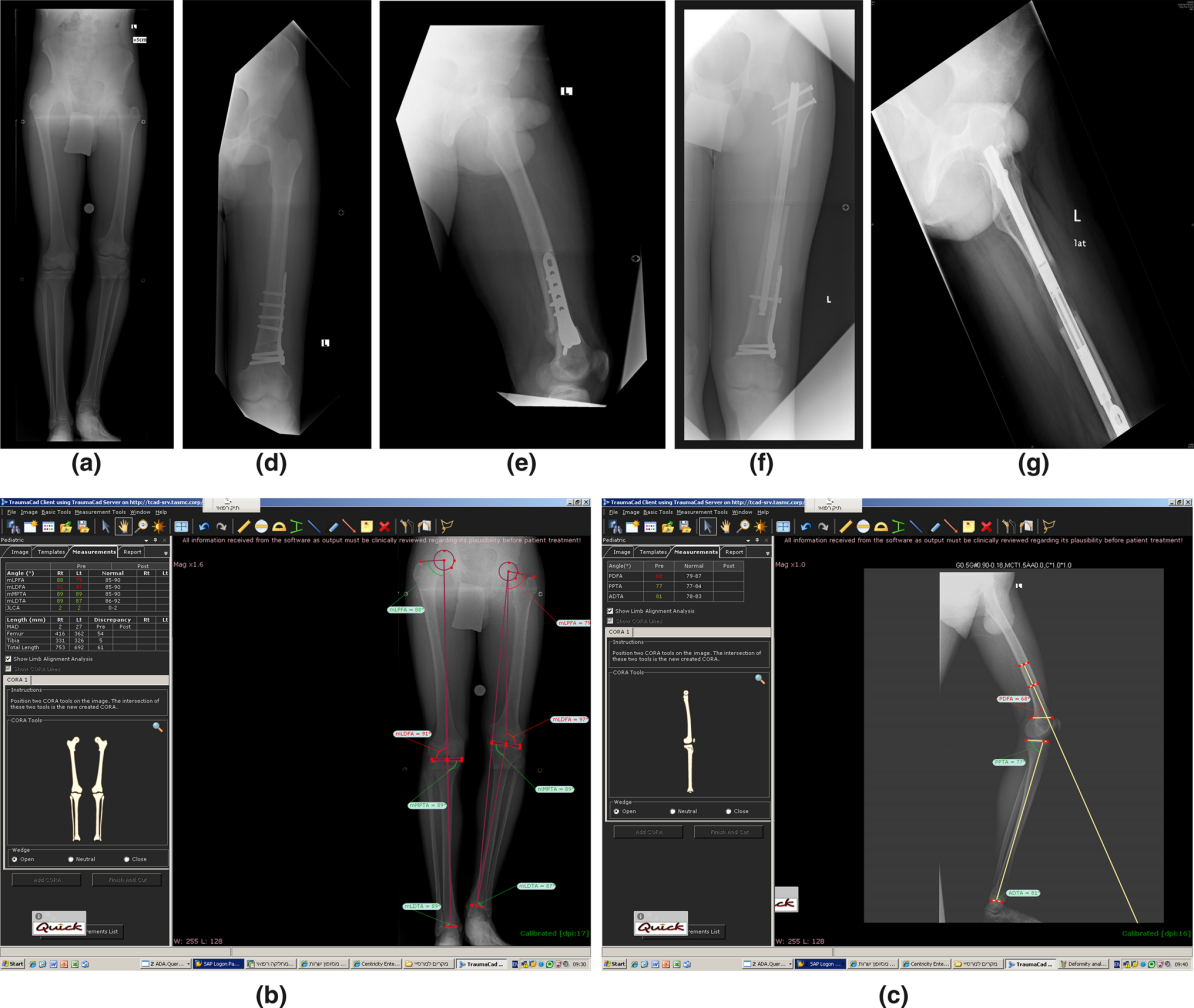
Case example 2

Male, aged 15.5 years, after a neonatal sepsis of the distal left femur with a growth arrest, presents with a shortening of 6.0 cm and a knee varus and procurvatum. He complains of knee pain (Fig. 7a).

The deformity analysis shows a left femoral shortening of 55 mm and a varus of the distal femur (Fig. 7b). There is also a femoral procurvatum of 20° (Fig. 7c).

### Considerations

The quality of the bone is good. There is a two-plane deformity of the distal femur. The shortening measures 55 mm.

We suggest performing the treatment in two stages: first to correct the deformity and second to lengthen the femur with an intramedullary nail.

During the first stage, a distal femoral osteotomy and plating is performed to correct the varus and the procurvatum (Fig. 7d, e).

Nine months later, during the second stage procedure, a gradual lengthening of the proximal femur is performed with a piriformis entry precise magnetic nail joined with the distal plate. The lengthening measures 55 mm (Fig. 7f, g).

## Conclusion

Successful deformity correction depends on establishing the aetiology of the pathology.

Clinical examination, additional laboratory tests and consultation with other experts may be needed to complete the workup.

Imaging studies include full-length standing X-rays in all relevant planes, and additional imaging modalities like computed tomography (CT) and magnetic resonance imaging (MRI) may add information on bone morphology and growth plates’ anatomy [3].

The data analysis of the deformity and length differences is performed and combined with the prediction at skeletal maturity.

The patient needs to be followed up on a regular basis, and repeat analysis should be done to improve the accuracy of the predictions.

Limb deformity and lengthening correction plan to achieve straight and equal lower limbs at maturity is made and updated during follow-up.

Timely surgical procedures are done using appropriate techniques and modern technologies [4, 5].
